# A Force-Activated Trip Switch Triggers Rapid Dissociation of a Colicin from Its Immunity Protein

**DOI:** 10.1371/journal.pbio.1001489

**Published:** 2013-02-19

**Authors:** Oliver E. Farrance, Eleanore Hann, Renata Kaminska, Nicholas G. Housden, Sasha R. Derrington, Colin Kleanthous, Sheena E. Radford, David J. Brockwell

**Affiliations:** 1 School of Molecular and Cellular Biology, Faculty of Biological Sciences, University of Leeds, United Kingdom; 2 Astbury Centre for Structural Molecular Biology, University of Leeds, United Kingdom; 3 Department of Biochemistry, University of Oxford, Oxford, United Kingdom; University of Washington, United States of America

## Abstract

Colicins are protein antibiotics synthesised by *Escherichia coli* strains to target and kill related bacteria. To prevent host suicide, colicins are inactivated by binding to immunity proteins. Despite their high avidity (K_d_≈fM, lifetime ≈4 days), immunity protein release is a pre-requisite of colicin intoxication, which occurs on a timescale of minutes. Here, by measuring the dynamic force spectrum of the dissociation of the DNase domain of colicin E9 (E9) and immunity protein 9 (Im9) complex using an atomic force microscope we show that application of low forces (<20 pN) increases the rate of complex dissociation 10^6^-fold, to a timescale (lifetime ≈10 ms) compatible with intoxication. We term this catastrophic force-triggered increase in off-rate a trip bond. Using mutational analysis, we elucidate the mechanism of this switch in affinity. We show that the N-terminal region of E9, which has sparse contacts with the hydrophobic core, is linked to an allosteric activator region in E9 (residues 21–30) whose remodelling triggers immunity protein release. Diversion of the force transduction pathway by the introduction of appropriately positioned disulfide bridges yields a force resistant complex with a lifetime identical to that measured by ensemble techniques. A trip switch within E9 is ideal for its function as it allows bipartite complex affinity, whereby the stable colicin:immunity protein complex required for host protection can be readily converted to a kinetically unstable complex whose dissociation is necessary for cellular invasion and competitor death. More generally, the observation of two force phenotypes for the E9:Im9 complex demonstrates that force can re-sculpt the underlying energy landscape, providing new opportunities to modulate biological reactions in vivo; this rationalises the commonly observed discrepancy between off-rates measured by dynamic force spectroscopy and ensemble methods.

## Abstract 2

A single-molecule force study shows that rapid dissociation of a high-affinity protein interaction can be triggered by site-specific remodelling of one protein partner, and that prevention of remodelling maintains avidity.

## Author Summary

Many proteins interact with other proteins as part of their function. One method of modulating the activity of protein complexes is to break them apart. Some complexes, however, are extremely kinetically stable and it is unclear how these can dissociate on a biologically relevant timescale. In this study we address this question using protein complexes between colicin E9 (a bacterial toxin) and its immunity protein Im9. These highly avid complexes (with a lifetime of days) must be broken apart for colicin to be activated. By using single-molecule force methods we show that pulling on one end of colicin E9 drastically destabilises the complex so that it dissociates a million-fold faster than its intrinsic rate. We then show that preventing this destabilisation (by the insertion of cross-links that pin the N-terminus of E9 in place) yields a kinetically stable complex. It has previously been postulated that force can destabilise a protein complex by partially unfolding one or more binding partners. Our work provides new experimental evidence that shows this is the case and provides a mechanism for this phenomenon, which we term a trip bond. For the E9:Im9 complex, trip bond behaviour allows a stable complex to be rapidly dissociated by application of a surprisingly small force.

## Introduction

Protein-protein interactions are integral to diverse cellular processes such as catalysis, transport, and signalling. For complexes of low affinity, changes in the relative concentrations of one (or more) binding partners, or alterations in the environment, are sufficient to trigger complex dissociation, allowing spatial and temporal control of the processes in question. More stable complexes require the input of chemical energy such as that provided by AAA+ proteins for their dissociation [1]. For high affinity complexes without direct access to an energy source, it remains unclear how dissociation can be induced on a biologically relevant timescale. This problem is exemplified by the rapid dissociation (lifetime≈minutes) of highly avid bacterial colicin:immunity protein complexes (K_d_≈10^−14^ M; lifetime≈days) upon binding to the outer membrane of target bacteria [2].

**Figure 1:**
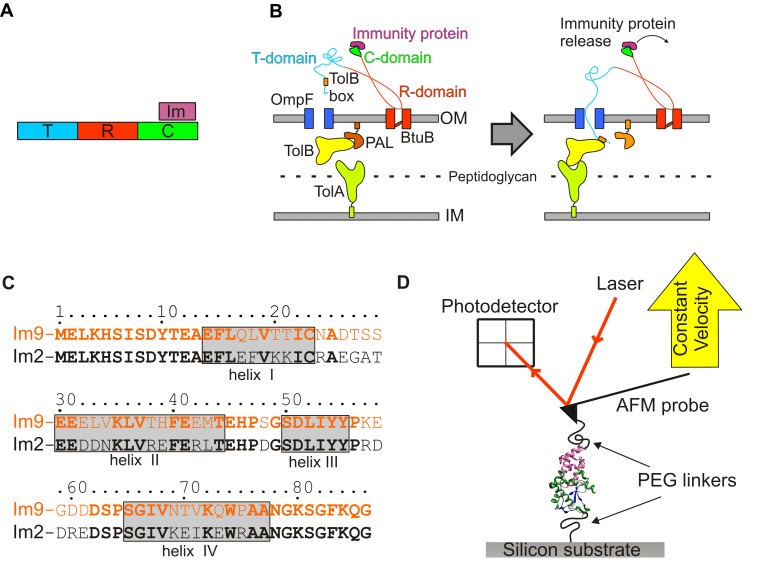
Summary of colicin toxicity, colicin nuclease:immunity protein interactions and experimental setup. (A) the domain structure of colicin E9 (from the N-terminus: translocation domain [T, light blue], receptor domain [R, red], and the nuclease domain [C, green]. Im9 (lilac) interacts exclusively with the nuclease domain. (B) Cartoon of the non-covalent interactions that are subverted by colicin E9 leading to *E.coli* cell death [2]. Left: intoxication is initiated by the binding of the R-domain of E9 to the BtuB receptor on the bacterial outer membrane (OM). Right: the intrinsically disordered T-domain of colicin E9 passes through the lumen of OmpF and binds to TolB via the TolB box motif, forming a quaternary “translocon” (E9:BtuB:OmpF:TolB) complex [58],[59]. Colicin E9 binding to TolB, enhances the affinity of TolB for TolA resulting in linkage between colicin E9 on the OM and the energised inner membrane (IM) [60]. Translocon-mediated contact with TolA induces dissociation of Im9 at the cell surface by an unknown mechanism that is PMF-dependent [12]. After Im9 release, the nuclease domain is translocated to the cytoplasm by an unknown mechanism. (C) Sequence alignment of Im9 (orange) and Im2 (black). Residues that are identical across Im9 and Im2 are shown in bold. The locations of α-helices I–IV are shown by grey rectangles. (D) Cartoon of the experimental setup. An immunity protein (lilac) and the nuclease domain of colicin E9 (green and blue) that both contain single, solvent accessible cysteine residues are immobilised onto the tip of an AFM cantilever or silicon substrate, respectively, via a hetero-bifunctional PEG linker (Figure S1). Upon retraction of the AFM tip at a pre-determined constant velocity the interaction strength can be quantified by measuring the displacement of the AFM tip via movement of a laser deflected onto a photodiode by the AFM tip.

**Figure 2:**
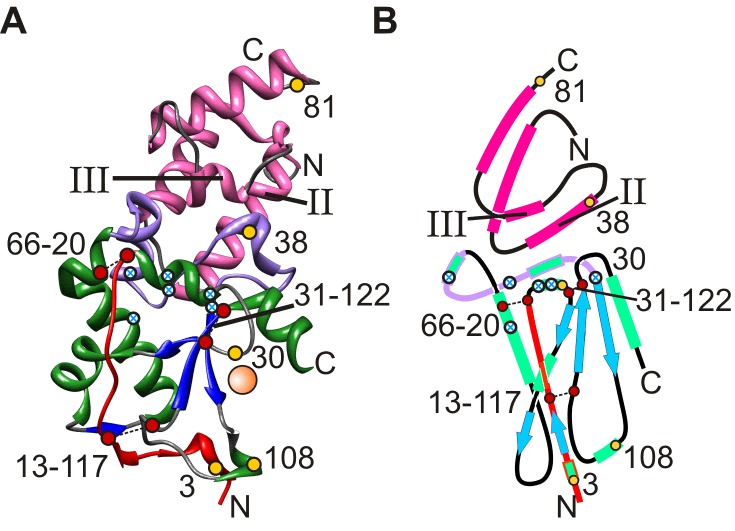
Structure of the E9:Im9 complex. (A) Crystal structure (PDB file: 1EMV) [5] of E9 DNase domain (green helices and blue β-strands) in complex with Im9 (pink). The metal ion binding site of the E9 DNase domain (orange sphere) denotes the active site. (B) topological cartoon of the complex. Helices are shown as rectangles (pink and green for Im9 and E9, respectively) and β-strands as blue arrows. The locations of single (yellow filled circles) or pairs (red filled circles linked by dashed lines) of cysteine residues used to immobilise each protein or cross-link E9 are shown for each structure. The locations of the affinity (III) and specificity (II) helices of Im9 are labelled in each structure. The N-terminal region (residues 1–20) and the Im binding surface of E9 (residues 72–98) are highlighted in red and lilac, respectively. Residues 23, 26, 58, 79, and 99 within E9 are discussed and are highlighted by white circles with blue crosses.

Colicins are protein antibiotics synthesised by *E.coli* strains to target and kill related bacteria during environmental stress [3]. The E-type colicins of group A, which include E2, E7, E8, and E9, exert their toxicity via nuclease activity. These multi-domain proteins (Figure 1A) contain a receptor domain (R) required for initial binding (to BtuB), a translocation domain (T) used to bring about translocation via interaction with OmpF and a cytoplasmic DNase domain (C, residues 480–582 of full length colicin, termed E9 herein) that results in death of the competing cell subsequent to translocation of this domain to the victim's cytoplasm (Figure 1B). To prevent host suicide, colicins are expressed alongside specific immunity proteins (Im2, Im7, Im8, and Im9) [2], which inactivate colicin enzymatic activity by binding to an exo-site adjacent to the active site (Figure 2A and 2B) [4]. The binding interface is typical for protein-protein complexes, covering a surface area of 1,575 Å^2^ for E9:Im9, which spans residues 72 to 98 of the nuclease domain [5]. The highly homologous cognate colicin:immunity protein pairs have high affinities (K_d_≈10^−14^ M) [6], while non-cognate pairs bind less tightly (e.g., for E9:Im2, K_d_≈10^−7^ M) [7]. The binding affinity of colicin:immunity protein complexes is determined by two binding “hotspots” on the immunity protein that interact with a distinct binding epitope on E9. Firstly for all colicin:immunity protein complexes, stabilising interactions are formed with residues in helix III of the immunity protein. This helix is identical in sequence in all E-type immunity proteins (apart from one residue in Im7 [6]). Residues within helix III of the immunity protein contact Phe86 and residues in the surrounding hydrophobic pocket of E9. This pocket comprises Tyr 83, Val 98, and the alkyl chains of Lys 89 and Lys 97 of the colicin DNase domain (Figure 2B, highlighted in lilac). Colicin:immunity protein affinity is modulated by stabilising (cognate complexes) or destabilising (non-cognate complexes) interactions between specificity-determining residues of helix II in the immunity protein (which differs significantly in sequence in different proteins; Figure 1C) and the binding interface of the colicin DNase domain (Figure 2) [8],[9]. As the on-rate for cognate and non-cognate colicin:immunity protein complexes is diffusion-limited (k_on_≈10^−8^ M^−1^s^−1^) [10],[11], the observed differences in affinity, which span almost ten orders of magnitude (K_d_ = 10^−14^ (E9:Im9) to 10^−4^ M (E9:Im7)) [7],[8],[11] are manifested in off-rates that differ by seven orders of magnitude (k_off_ = 10^−6^ to 10^1^ s^−1^ for the cognate and non-cognate complexes, respectively).

The large differences (10^7^) in off-rates of different colicin:immunity protein complexes render this system an excellent model for investigating the molecular determinants of molecular recognition and, in particular, for exploring how highly avid complexes can be remodelled in vivo in the absence of an external energy source to allow rapid dissociation when required for biological activity. While the precise mechanism of E-type colicin:immunity protein dissociation is unclear, it is known that colicin invasion uses molecular mimicry to subvert a series of protein-protein interactions that result in linkage of the colicin (bound to the outer membrane) to the TolQRA complex of the energised inner membrane (a translocon; Figure 1B). As TolQRA function and colicin intoxication both require a proton gradient across the inner membrane [12], it has been postulated that the energy of the proton motive force (PMF) may be harnessed to drive colicin:immunity protein dissociation, a necessary prerequisite of translocation of the nuclease to the cytoplasm.

“Inside-out” energy transduction mechanisms are exemplified by the Ton system, which is highly homologous to TolQRA (both require a functioning PMF to carry out their function [13]). In the case of the Ton system, PMF-driven remodelling of the plug domain of the outer membrane protein BtuB allows siderophore import. Remodelling is also thought to play a role in E9 colicin intoxication as cross-linking residues 20 and 66 of the nuclease domain prevents insertion into planar lipid bilayers and protects against cellular toxicity [14]. In accord with a requirement for structural remodelling in the mechanism of colicin invasion, immunity protein release that is usually triggered by formation of the translocon in the presence of the PMF, is prevented by cross-linking of the N- and C- termini of the R-domain (Figure 1B) [15].

Here we use single molecule force methods (using atomic force microscopy) to investigate the requirement for structural remodelling in the dissociation of single E9:immunity protein complexes under defined rates of loading and pulling geometry. The effects of structural re-arrangements in proteins can be investigated by many approaches, but most apply a “peturbant” globally. Single molecule force methods (which use mechanical extension as a peturbant) are ideally suited for such an investigation as force is applied locally to the complex at positions determined by the sites of linker attachment. Using this approach, we show here that a low level of force (<20 pN) commensurate with that applied by protein molecular motors [16]–[18] increases the dissociation rate of the E9:Im9 complex in vitro by a remarkable 10^6^-fold. Using mutagenesis and disulfide cross-linking, we also elucidate the force transduction path through E9, which catalyses complex dissociation, and show that this involves conformational remodelling of E9 triggered by mechanical deformation of its terminal region. The data show that mechanical force can be exploited to enable rapid dissociation of the high affinity colicin:immunity protein interaction by application of force at the N-terminus of E9.

## Results

### Measuring the Dynamic Force Spectrum of Colicin:Immunity Protein Complexes

**Figure 3:**
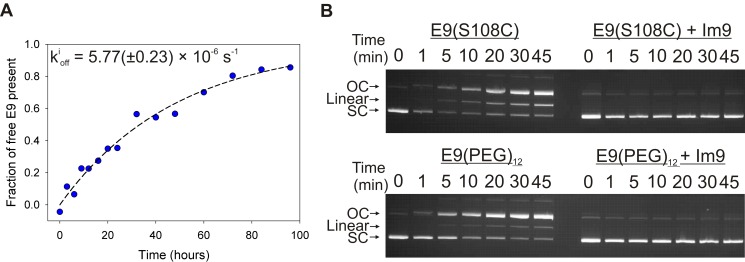
Effect of PEG derivatisation on the dissociation rate of E9:Im9 and activity of E9. (A) Determination of the ensemble dissociation rate constant (k^i^_off_) for E9 (S3C):Im9 (S81C) derivatised with methyl-(PEG)_12_-maleimide (Materials and Methods; Figure S8). (B) E9 (S108C) DNase activity assay (Materials and Methods) before and after derivatisation with methyl-(PEG)_12_-maleimide. Left: in the absence of Im9, a decrease in supercoiled (SC) and a corresponding increase in the intensity of linear and open circular (OC) DNA is observed over time, indicating nuclease activity. Right: addition of a 1 .7 molar excess of Im9 ablates this activity.

**Figure 4:**
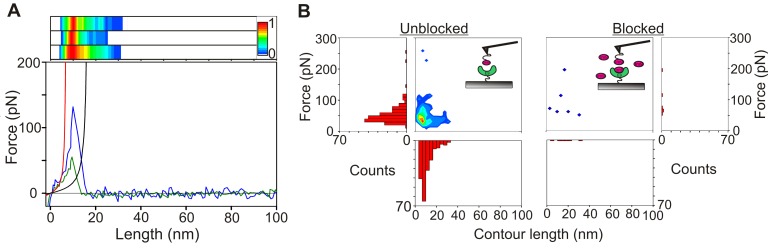
Quantifying unbinding forces for E9:immunity protein complexes. (A) Bottom: typical force extension profiles for E9:Im9 dissociation for 3:81 (green) and 3_20–66_:81 (blue) both taken with retraction velocities of 1,000 nms^−1^. Solid black line corresponds to a worm-like chain (WLC) with a persistence length of 0.4 nm and a contour length of 17.96 nm for an interaction between Im9 immobilised via linkers to the tip apex and E9 immobilised to a substrate via linkers directly below the tip. Solid red line shows a WLC with the most probable contour length after modelling for tip effects (Text S1) where the maximum expected contour length used in the simulation is 17.96 nm. Top: frequency distribution of fitted contour lengths of unbinding events from unfiltered data. The normalised frequency of a particular contour length is displayed as a colour scale from 0 (white) to 1 (red). Each plot (E9:Im9 3:81 for a dataset accumulated at 500, 5,000, or all retraction velocities, top to bottom) shows a single maximum centred on 10 nm. (B) Contour plot and force and contour length frequency histograms of unfiltered data showing that the force and extension at which E9:Im9 unbinding occurs are highly correlated and yield a single unbinding event. Addition of excess Im9 to the solution during measurement ablates these correlated events.

We used atomic force microscopy to measure the dynamic force spectrum of the unbinding of single complexes of the nuclease C-domain of colicin E9 bound to its cognate immunity protein (Im9), together with non-cognate complexes of E9:Im2 and several variants of Im9 containing point mutations in the binding site [7],[8]. The experimental design is depicted in Figures 1D and S1. Briefly, single cysteine residues were introduced into E9 (the wild-type protein lacks cysteine residues) and pseudo–wild-type variants of Im9 and Im2 in which the single naturally occurring cysteine was first mutated to alanine (C23A), to enable immobilisation of each protein specifically to the substrate or cantilever. Sites chosen for mutation to allow immobilisation (S3C, S30C, or S108C in E9 and T38C or S81C in the immunity protein) were solvent accessible and distal to the E9:immunity protein binding interface (Figure 2A and 2B). The immunity protein and E9 were next attached to the atomic force microscope (AFM) tip and substrate, respectively, using hetero-bifunctional polyethylene glycol (PEG) linkers of variable length (Materials and Methods; Figure S1). Gel filtration was used to compare the ensemble off-rates of the wild-type complex and one containing mutated E9 derivatised with PEG linker (k^i^_off_ = 1.8×10^−6^ s^−1^ and 5.8×10^−6^ s^−1^ for wild-type [8] and derivatised [E9 S3C:Im9 (S81C)] complexes [Figure 3A], respectively). These data, together with a nuclease assay (Materials and Methods; Figure 3B) showed that neither sequence changes nor PEG derivatisation significantly affected the properties of E9 alone or in complex with Im9. Complexes were repeatedly formed and dissociated by approach-retract cycles of the functionalised AFM tip towards and away from the surface at a defined velocity (Materials and Methods). Unbinding resulted in a single force peak characteristic of a single molecule unbinding event (Figures 4A, bottom, lower plot, and Figure S2) for 99.5% of all force-extension profiles that showed any evidence of interaction between the tip and substrate (typically 10% of all approach-retract cycles). All force-extension data are presented and analysed after accounting for the deflection of the AFM tip (i.e., the distance between apex of the AFM tip and the substrate surface).

Initially, force-extension profiles that displayed a detectable unbinding event greater than 5 nm from the surface (to avoid non-specific tip-surface interactions, see Text S2) were analysed without further filtering. Figure 4B shows a scattergram contour plot and individual frequency histograms for the unbinding force and contour length at rupture for every unbinding event in each force-extension profile of a single dataset. These data show that unbinding events occurred over a narrow range of extensions (mode = 10 nm) suggesting that unbinding occurs by a single pathway (Figure 4A, top). Interestingly, the measured contour length is significantly shorter than that expected (Figure 4A bottom, black solid line) based on the sum of the lengths of each linker (6.62 nm each) and the through space distance between the extension points on E9 and Im9. This distance is 4.72 nm when extending the complex from residue 3 on E9 and residue 81 on Im9. This complex is denoted 3:81 (similar nomenclature is used throughout). To predict the expected contour length more accurately, it is necessary to account for the ability of Im9 to be immobilised anywhere between the apex and the base of the AFM tip and the distribution of end-to-end lengths within the ensemble of the polymeric linkers (Figure S3). Taking these effects into consideration (using a Monte Carlo simulation; Figure S4 and Text S1) yielded a contour length significantly shorter than observed (7.57 nm, Figure 4A bottom, red line). Importantly, these calculations suggest that the complex undergoes deformation or elongation prior to dissociation (see Discussion).

In order to quantify the unbinding forces and loading rates at rupture, force-extension profiles were subsequently analysed using an automated analysis script whereby events (<13% of total force-extension profiles; ) were filtered from non-specific interactions on the basis of their force-extension profiles. To be binned for further analysis (Text S2) a force-extension profile was required to (i) have a rupture force larger than the thermal noise of the experiment (18 pN; Figure S5 and Text S3); (ii) have a distance to the rupture event from the hard tip-surface contact that was between 5 and 32.5 nm or 5 and 40 nm for protein complexes immobilised using (PEG)_4_ and (PEG)_12_, respectively. The lower limit avoids the analysis of any non-specific tip-sample interactions and the upper limit is significantly greater than the expected rupture distance so that all events are analysed; and (iii) display only a single unbinding event.

The ability of the experimental setup and data analysis method to recognise only specific E9:Im9 unbinding events was verified using two controls. Firstly, the addition of excess immunity protein to the solution between the AFM tip and substrate resulted in a decrease of the frequency of unbinding events from more than 12% to less than 1% (Figure 4B). Secondly, the addition of EDTA was found to decrease the event frequency 3-fold. EDTA sequesters divalent metal cations from E9, destabilising the protein substantially (T_m_ = 36°C and 68°C for apo- and zinc-bound E9 [19]) leading to a loss of binding to Im9. Addition of excess Zn^2+^ restored E9 stability and, consequently, event frequency (unpublished data).

The force and loading rate at unbinding of E9:Im9 were measured for each event from force-extension data (Text S2). Force-frequency distributions (Figure S6) were subsequently calculated for each dataset (typically 100–200 events; Table S1), allowing the extraction of the most probable unbinding force (Figure S6; Text S4) and the loading rate at rupture. The dynamic force spectrum of each complex was then revealed by quantifying how the force at rupture varies as a function of the force loading rate between 700 and 180,000 pNs^−1^ (Text S5).

### Forced Unbinding of the E9:Im9 Complex Occurs by a Two-Stage Mechanism

**Figure 5:**
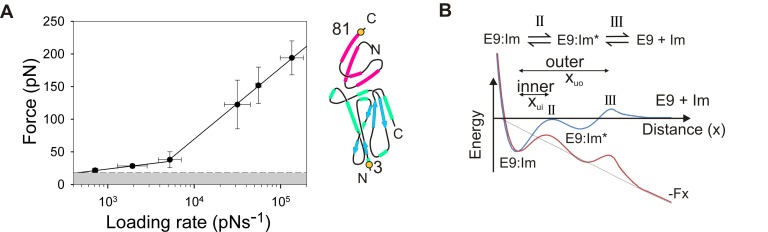
Measuring the dynamic force spectrum for E9:Im9. (A) The dynamic force spectrum (a plot of the unbinding force versus the natural logarithm of the loading rate) of E9:Im9 3:81 reveals a three state transition. Error bars are based on the standard deviation of measurements from triplicate datasets taken at each retraction velocity. Forces below the detection limit of the instrument and filtering software (18 pN, dashed line) are coloured grey. The pulling positions (3:81, yellow filled circles) relative to the binding interface is shown schematically alongside. (B) Postulated mechanism (top) and energy landscape (bottom) for the (un)binding of E9 from immunity proteins. An initial encounter complex (E9:Im*) is formed followed by the formation of the bound complex (E9:Im). Application of force tilts the energy landscape (by –Fx) so that barriers that are “invisible” in the absence of force (blue landscape) become rate limiting in the presence of force (red landscape).

The dynamic force spectrum of E9:Im9 dissociation was initially measured by immobilising E9 close to the N-terminus (residue 3) (Figure 2) as this region is immediately adjacent to the R-domain and contiguous with the T-domain, which is translocated during colicin intoxication in vivo (Figure 1B). No force is likely to be applied directly to the immunity protein in vivo. A pulling location was thus selected for Im9 (residue 81) (Figure 2) that is solvent exposed and distal to the binding interface. Sample force-frequency histograms that span the range of loading rates used (700–180,000 pNs^−1^) and the resultant dynamic force spectrum for this complex (3:81) are shown in Figures S6 and Figure 5A, respectively. Two force regimes are evident. At low loading rates (<5,400 pNs^−1^), dissociation occurs at low forces with a shallow dependence of unbinding force on the loading rate. This allows rapid dissociation of an avid complex at biologically accessible loading rates [16],[17],[20], or by application of biologically accessible forces (see [16]–[18] and references therein). For example, at a force of 20 pN, the lifetime of E9:Im9 is approximately 12 ms, in contrast to 4.1 d in the absence of force. At higher loading rates (>5,400 pNs^−1^) the complex is highly force resistant and the unbinding force is strongly dependent on the loading rate. The simplest explanation for these observations is that unbinding occurs by a three-state mechanism: at low forces unbinding rates are limited by a barrier in the energy landscape distal to the bound ground state of the complex (a large x_u_; Figure 5B, x_uo_). At higher forces, tilting of the energy landscape results in a previously hidden inner barrier (a small x_u_) becoming rate limiting (Figure 5B, x_ui_). Such a mechanism is consistent with the dual-recognition (un)binding pathway for E9:Im9 determined using ensemble fluorescence experiments (Figure 5B, top) [8]. In this mechanism, the affinity of the initial encounter complex is determined by interactions between residues of the E9 binding interface (Figure 2, highlighted in lilac) and helix III of the immunity protein (residues S50, D51, I53, and Y55) [5]. Rigid body rotations of the initial encounter complex then allow the formation of stabilising (cognate) or less stabilising (non-cognate) interactions between E9 and specific residues in helix II of the immunity protein [7]. Accordingly, the outer barrier measured by DFS that is rate determining at low rates of forced unbinding is expected to report on the free energy difference between the native state and the barrier for dissociation of E9 from helix III of Im9, while the inner barrier that is rate determining at high rates of forced unbinding is expected to report on the energy gap between the native state and the barrier for dissociation of E9 from helix II (Figure 5B).

To confirm the apparent similarity between the force- and thermally activated unbinding mechanisms of E9:Im9, each linear region of the dynamic force spectrum was fitted to the Bell-Evans equation [21] (Equation 1, where f* is the most probable unbinding force, *r*_f_ is the force loading rate at rupture, *T* is the temperature, and *k*_B_ is Boltzmann's constant). This allows the dissociation rate constants *in the absence of force* (k^0F^_off_) and the “distance” along the free energy landscape from the bound state to the barrier that is rate limiting for dissociation (x_u_) to be obtained (Table S1).
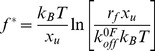
(1)Obtaining k^0F^_off_ values by this method assumes that the outer barrier observed in the dynamic force spectrum remains rate-limiting at lower loading rates that are inaccessible to this technique (see [22],[23] for example). These parameters were found to be k^0F^_off_ = 50±17 s^−1^, x_u_ = 0.9±0.2 Å, and k^0F^_off_ = 4.9±1.3 s^−1^, x_u_ = 5.8±0.4 Å for the inner and outer barriers of E9:Im9 dissociation, respectively. If forced unbinding (at the loading rates applied in these experiments) occurs over the same energy landscape as for the thermally induced pathway, the extrapolated k^0F^_off_ for the outermost barrier (the rate determining step at low force), determined by DFS should be identical to that measured by ensemble methods (k^i^_off_) [22],[24]–[28]. Remarkably, forced unbinding of E9:Im9 results in a k^0F^_off_ that differs from k^i^_off_ by a striking six orders of magnitude (≈10^0^ and 10^−6^ s^−1^, respectively).

**Figure 6:**
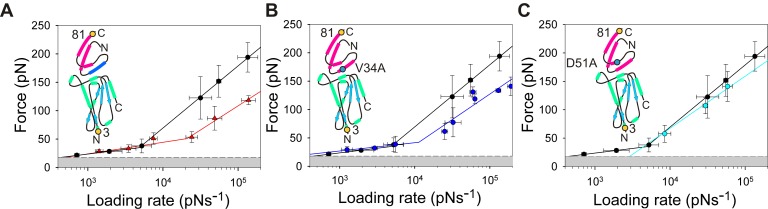
Assigning the dynamic force spectrum of E9:Im dissociation. The dynamic force spectrum of (A) E9:Im2 (D33A) (red), (B) E9:Im9 (V34A) (blue), and (C) E9:Im9 (D51A) (cyan) are compared with E9:Im9 (all graphs, black). Error bars are based on the standard deviation of measurements from triplicate datasets taken at each retraction velocity. Forces below the detection limit of the instrument and filtering software (18 pN, dashed line) are coloured grey. The pulling position (3:81, yellow filled circle) relative to the binding interface is shown schematically alongside. The location of the altered specificity helix II of Im2 (D33A) (A, blue rectangle) and point mutations (B and C, blue filled circle) are highlighted.

**Figure 7:**
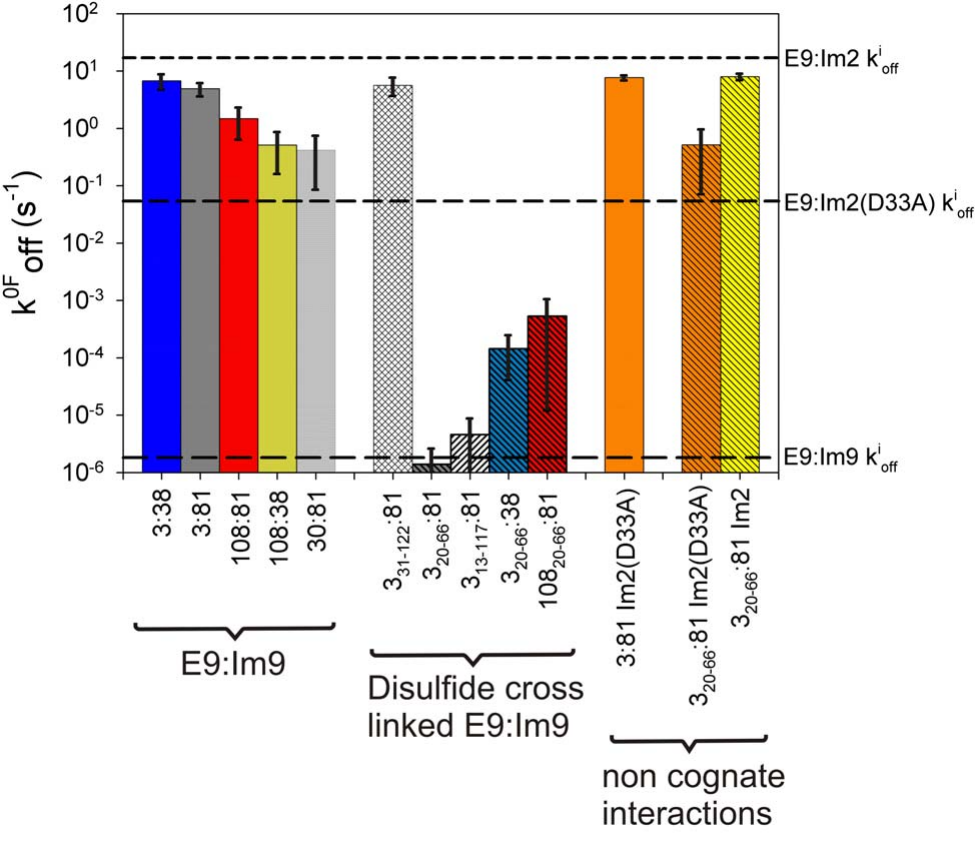
Summary of DFS measurements. Comparison of k^0F^_off_ values (bars) obtained from DFS data compared with values obtained by bulk phase experiments (k^i^_off_, dashed lines) for the same interaction. Errors were calculated by a Jackknife method.

To examine whether the rapid rate of force-induced dissociation of E9:Im9 is observed for other E9:immunity protein complexes, the dynamic force spectrum of a non-cognate complex (E9:Im2 (D33A), k^i^_off_ = 0.054 s^−1^) [7] was next examined (Figure 6A). This variant was selected since it has a higher affinity for E9 compared with wild-type Im2 (K_d_ = 1×10^−9^ M and 1.5×10^−7^ M, respectively) [7]. Again two force regimes were observed in the dynamic force spectrum of this complex, each of which has a similar x_u_ value to that observed for each barrier of the cognate E9:Im9 complex. This indicates that force-induced unbinding of the cognate and non-cognate complexes occurs by a similar three-state mechanism. At low loading rates that probe the rate determining outer barrier, the unbinding forces (and thus k^0F^_off_) were closely similar for the cognate and non-cognate complexes (Figure 6A; Table S1). Under force, E9:Im2(D33A) thus behaves identically to E9:Im9 (Figure 7, solid dark grey and orange bars, respectively), despite k^i^_off_ values that differ by four orders of magnitude. Similar to the behaviour of E9:Im9, k^0F^_off_ for E9:Im2(D33A) is also faster than its known k^i^_off_ (7.6 s^−1^ versus 0.054 s^−1^) [7], indicating that the underlying energy landscape for immunity protein dissociation from E9 is highly sensitive to the effects of force, regardless of the nature of the bound immunity protein.

By contrast with the dynamic force spectrum of colicin:immunity proteins at low loading rates (governed by the outer barrier), the inner barrier for dissociation of E9:Im2(D33A) is reduced significantly compared with that of the cognate complex and only becomes visible at loading rates >22,000 pNs^−1^ (Figure 6A). In these kinetic unbinding experiments each force regime is assumed to probe the free energy difference between the bound state and each barrier to unbinding. As the energy difference between the bound and free states is reduced for E9:Im2(D33A) relative to E9:Im9 (K_d_ is reduced 10^5^-fold [7]), a reduction in unbinding force would be expected for both the inner and outer barriers. Instead, application of force at residue 3 of E9 appears to decouple the dual recognition sites of helices II and III of the immunity protein with E9. The inner barrier measures the “strength” of the specificity residues in helix II of the immunity protein and E9 (which are stabilising for Im9 and less stabilising for Im2(D33A); Figure 1C), while the outer barrier height is determined by the stability of interactions between E9 and immunity protein helix III (identical in sequence across all DNase E-colicins except a Thr substitution for Ser at position 51 of Im7) [6].

As discussed above, the inner and outer barriers appear to be due to the dissociation of immunity protein helices II and III, respectively, from the binding surface of E9. To confirm this assignment, DFS was used to measure E9 unbinding from Im9 variants containing single point mutations that destabilise either the cognate specificity interactions of the inner barrier (V34A in helix II), or interactions that define the outer barrier (D51A in helix III) [8] (Figure 6B and 6C). As predicted, unbinding forces for E9:Im9(V34A) were identical to those for wild-type E9:Im9 at loading rates <5,400 pNs^−1^, but were reduced by more than 35 pN compared with E9:Im9 at loading rates higher than this (x_u_ remained constant for both barriers). By contrast, at loading rates <3,000 pNs^−1^, the unbinding forces for E9:Im9(D51A) were reduced to a level below the thermal noise limit of the instrument. However, at higher loading rates, E9:Im9(D51A) behaves similarly to the wild-type E9:Im9 complex. These data are consistent with the proposed dual-site recognition process for colicin:immunity protein (un)binding [8] with force effectively uncoupling the unbinding of helices II and III of Im9 from the E9 binding surface. The ability to assign each regime of the dynamic force spectrum to the unbinding of the two recognition sites of the complex previously identified by ensemble methods [8] renders the presence of an additional hidden barrier unlikely. If present, a hidden barrier would require an x_u_ value of greater than 3.7 nm to obtain a k^0F^_off_ value commensurate with k^i^_off_. This is larger than the outermost barrier previously observed for the dissociation of biotin from avidin [22]. In contrast to the characteristically flat recognition surface of E9:Im9 that is typical of protein-protein interactions in general, biotin resides in a deep pocket within avidin. We thus consider the presence of an additional barrier unlikely. Overall, therefore, the results indicate that the application of force distal to the E9:Im9 interface enables rapid dissociation of this tight binding complex such that the dissociation rate is enhanced by greater than a million-fold to a timescale commensurate with the kinetics of cell killing by colicins (within minutes) [2],[15],[29].

### Complex Affinity Is Modulated by Pulling Geometry

**Figure 8:**
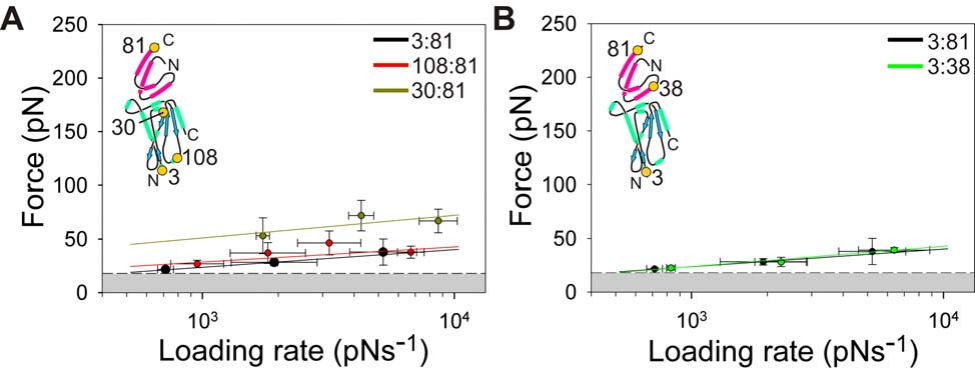
The effect of pulling geometry on the dynamic force spectrum of E9:Im dissociation. (A) Dynamic force spectrum of E9:Im9 when extended from residues 3 (black circles and line), 30 (ochre circles and line), and 108 (red circles and line) on E9 and residue 81 on Im9. (B) Dynamic force spectrum of E9:Im9 when extended from residue 3 on E9 and residue 38 (green circles and line) or 81 (black circles and line) on Im9. Error bars are based on the standard deviation of measurements from triplicate datasets taken at each retraction velocity. Forces below the detection limit of the instrument and filtering software (18 pN, dashed line) are coloured grey. The pulling positions (yellow filled circles) relative to the binding interface is shown schematically (inset). For clarity, only the outer barrier that is rate limiting at low loading rates is plotted.

Force induced conformational changes are known to trigger catalysis [30] or expose “cryptic” binding sites [31] in some proteins. These remodelling events are usually very sensitive to the points of force application as proteins are known to display anisotropic force responses. Thus, when extended in different directions proteins can appear to be mechanically strong or weak [32]–[35]. To investigate whether this effect is the origin of the force-induced lability of E9:immunity protein complexes, the effect of altering the pulling location on the dynamic force spectrum of the E9:Im9 complex was examined. Accordingly, different residues on E9 and Im9 (positions 3, 30, and 108 on E9 and positions 38 and 81 on Im9) were mutated individually to Cys to enable immobilisation to the surface at different points (Figure 2). These experiments showed that k^0F^_off_ for the outer (rate-limiting) barrier remains 10^5^- to 10^7^-fold higher than k^i^_off_ regardless of the pulling location employed (Figures 7 and 8; Table S1). Nonetheless a small, but significant, dependence of the unbinding force (Figure 8A) and k^0F^_off_ (Figure 7) on the immobilisation site on E9 was observed, with the highest k^0F^_off_ values occurring when E9 was pulled from an N-terminal location (residue 3, k^0F^_off_ = 4.9 s^−1^) and lower values occurring when E9 was immobilised at position 108 or 30 (k^0F^_off_ = 1.5 and 0.4 s^−1^, respectively; Figure 7 and Table S1). By contrast, k^0F^_off_ was insensitive to the pulling location on Im9 (Figure 8B). The anisotropy in k^0F^_off_ in relation to the E9 pulling location, together with the large disparity between k^0F^_off_ and k^i^_off_ values and the increase in chain length upon dissociation being greater than expected based on linker length (Figure 4A), suggest that remodelling or partial unfolding of E9 takes place under force. This then yields a dissociation pathway with a smaller activation free energy than is accessible in the absence of remodelling. The ability to alter the unbinding kinetics by force-induced substrate remodelling has recently been postulated [36],[37]. The results presented here show a striking example of this phenomenon, with rate enhancements of a million-fold caused by application of only 20 pN force, at most.

### Disulfide Cross-Links Decrease Forced Unbinding Rates

**Figure 9:**
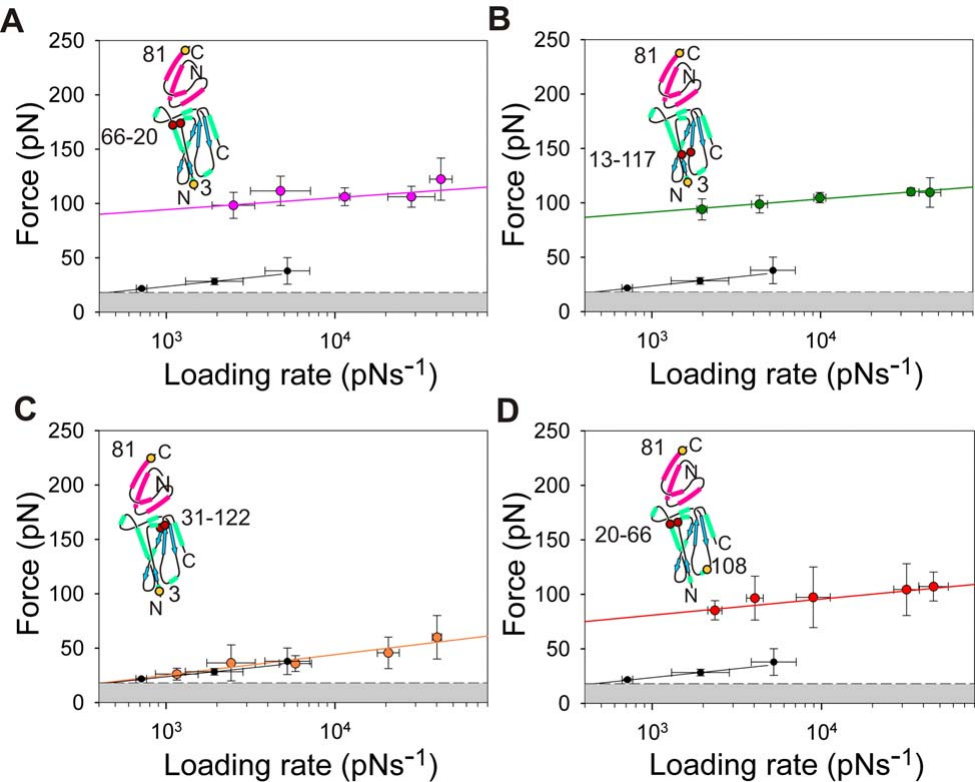
Dynamic force spectra of cross-linked variants of E9. When extended between residues 3:81, insertion of a disulfide cross-link close to the N-terminus of E9 (between residues 20 and 66) (A, pink circles and line) or 13 and 117 (B, green circles and line) yields a force resistant complex (k^(0F)^_off_ = 1.4±1.2×10^−6^ s^−1^ and 4.6±4.2×10^−6^ s^−1^, respectively). By contrast, insertion of a disulfide cross-link between residues 31 and 122 has no protective effect and yields a dynamic force spectrum (C, orange circles and line) identical to the outer barrier observed for wild-type E9:Im9. The protective effect of cross-linking residues 20 and 66 is partly nullified by moving the position of force application onto E9 from residue 3 to residue 108 (D, red circles and line). The dynamic force spectrum of the outer barrier of uncross-linked E9:Im9 3:81 (black data points and line) is shown in all graphs for reference. Error bars are based on the standard deviation of measurements from triplicate datasets taken at each retraction velocity. Forces below the detection limit of the instrument and filtering software (18 pN, dashed line) are coloured grey. The pulling positions (yellow filled circles) and positions of the disulfide cross-links (red filled circles) relative to the binding interface are shown schematically for each experiment (inset).

The data described above demonstrate that the level of acceleration in the dissociation rate of E9 from Im9 is sensitive to the precise location of force application and that the rate enhancement is greatest when force is applied close to the N-terminus of E9. Examination of the structure of E9 shows that the N-terminal 30 residues (highlighted in red, Figure 2B) do not contact the immunity protein binding interface directly. Leu 23 and Ala 26 of the N-terminal region of E9 do, however, form part of hydrophobic core of E9 formed around Trp 58 that also encompasses residues of the binding interface (Val 79, Pro 85, and Tyr 99) (Figure 2B). The N-terminal region of E9 may thus relay the force trigger to an allosteric site of affinity modulation. To understand the signal transduction pathway in more detail, and to identify the location of the allosteric site that translates the mechanical stimulus to an increase in dissociation rate, a series of mutant E9 domains were produced containing disulfide bonds in different locations of the protein structure (Figure 2A and 2B). Disulfide bond formation in all of these variants was shown to be spontaneous and to proceed to completion using Fourier transform ion cyclotron mass spectrometry (Figure S7). The dynamic force spectrum of E9:Im9 complexes extended from the N-terminal region of E9 (3:81) engineered to contain a disulfide bond that links the N-terminal region of the polypeptide chain to the remainder of the folded globular region of E9 (linking residues 13–117 or 20–66; Figure 2) are shown in Figures 9A and 9B, respectively. Remarkably, both of these cross-linked E9 variants display a simple monotonic dynamic force spectrum over the entire accessible loading rate range with significantly increased unbinding forces (ΔF≈100 pN relative to wild-type complexes). The value for x_u_, however, is similar to that observed for the outer barrier in the dynamic force spectrum of the uncross-linked, wild-type E9:Im9 complex. Fitting these data to the Bell-Evans model yields k^0F^_off_ values for these complexes that are increased by ≈10^6^-fold, resulting in values for k^0F^_off_ that are similar to those measured using ensemble techniques (k^0F^_off_ = 4.6×10^−6^ s^−1^ and 1.4×10^−6^ s^−1^ for 3_13–117_:81 and 3_20–66_:81, respectively; Figure 7). Values of k^i^_off_ measured using gel filtration experiments under identical conditions to those employed for the AFM experiments are 3.0×10^−6^ and 5.8×10^−6^ s^−1^ for E9_20–66_:Im9 and pseudo wild-type E9:Im9 derivatised with methyl-(PEG)_12_-maleimide, respectively (Materials and Methods; Figure S8; Table S1). Addition of 4 mM DTT reversed this mechanical strengthening, leading to unbinding forces identical to those of wild-type E9:Im9 (Figure S9). To localise the region of E9 involved in force remodelling more precisely, the dynamic force spectrum of a third E9:Im9 (3:81) complex that contains a disulfide cross-link distal to the N-terminal region of E9 (31–122; Figure 2B) was analysed. In this case no force enhancement was observed. Instead, a dynamic force spectrum with a single force regime was obtained, identical to that of the outer barrier of the wild-type uncross-linked complex (Figure 9C). These data localise the allosteric trigger to residues 21–30 or to residues 118–121 in E9. As E9 is extended from the N-terminus in these experiments we consider the latter site to be unlikely as the site of the trigger.

The data presented here reveal that insertion of a disulfide bond is able to modulate how force is propagated through the nuclease domain of colicin E9 preventing remodelling of E9 and thus facile complex dissociation. Extension of the complex in a geometry that propagates the force via a different path would thus be expected to render the cross-link between residues 20 and 66 less effective. In accord with this hypothesis, k^0F^_off_ values for E9_20–66_:Im9 complexes were found to be dependent on the position at which force is applied to both E9 and Im9 (k^0F^_off_ = 4.0×10^−5^ s^−1^, 1.4×10^−6^ s^−1^, and 5.3×10^−4^ s^−1^ for 3_20–66_:38, 3_20–66_:81, and 108_20–66_:81, respectively; Figures 7 and 9D; Table S1).

**Figure 10:**
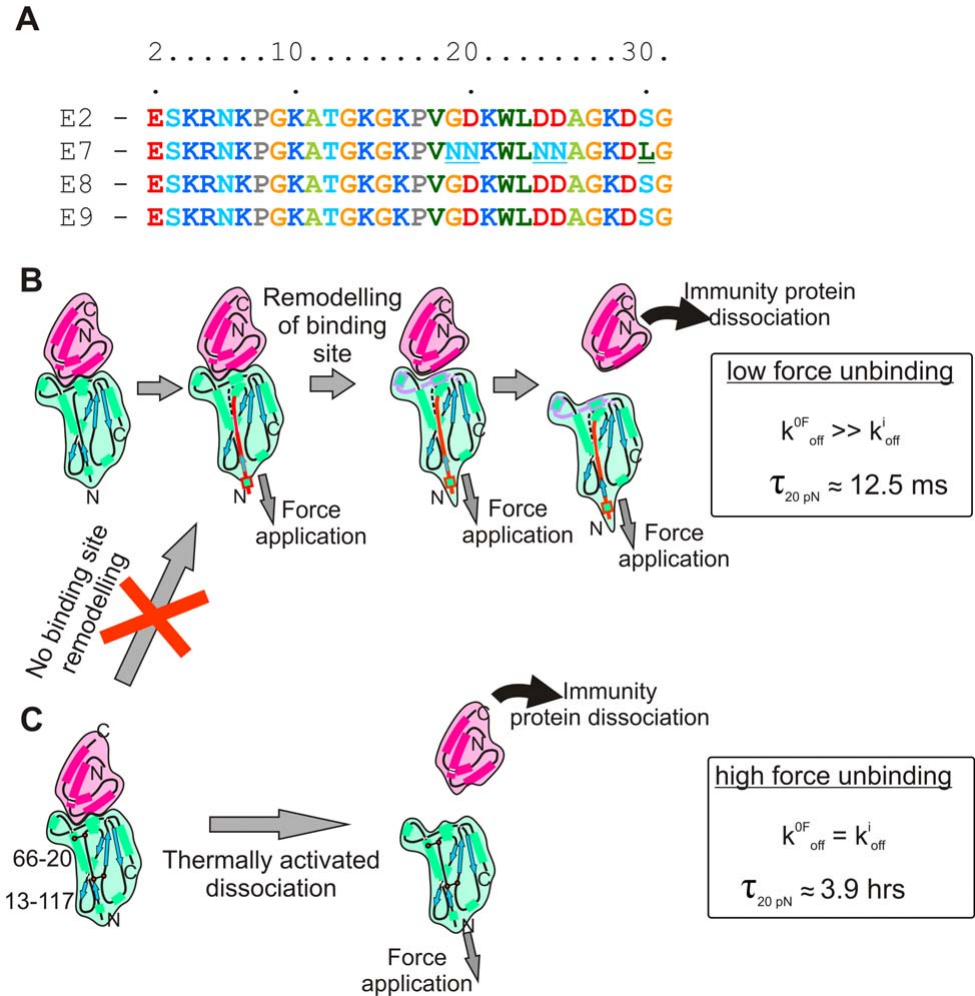
Summary of E9:Im9 dissociation under applied force. (A) Comparison of N-terminal residues of the C-domain of a number of E type colicins from group A showing that this region is highly conserved. Residues are numbered for the isolated domains rather than the intact colicin sequence for clarity. Residues are coloured by amino-acid. (B) Application of forces below the sensitivity of the experimental setup used in this work (<18 pN) lead to rapid immunity protein dissociation where k^0F^_off_ is ≈10^6^-fold greater than the corresponding ensemble rate measurements for k^i^_off_. (C) Introduction of disulfide bonds in the N-terminal region of E9 results in k^0F^_off_ values that agree with k^i^_off_ measurements. The data presented are consistent with a high affinity protein-protein interaction that has evolved to dissociate rapidly under the application of an appropriate trigger force.

The sensitivity of k^0F^_off_ to the pulling location on E9 and to the presence of cross-links in the N-terminal region of this protein demonstrates that force can act as an allosteric trigger for E9:Im9 complex dissociation. We have identified residues 21–30 as the most probable location of this trigger, a region that both links to the N-terminus and contacts residues involved in the binding interface. To be an effective transducer of mechanical signals, the N-terminus of E9 (residues 1–20) would be expected to be mechanically labile. Analysis of the sequences of colicin E2, E7, E8, and E9 reveal that the N-terminal region of all four nuclease domains is highly conserved and has a high content of small aliphatic amino-acids (RNKP**G**K**A**T**G**K**G**KP**V**G**D**; Figure 10A). This region of the protein thus docks against the remainder of the globular domain with little side-chain interdigitation, commensurate with the requirements of a trigger activated at low forces [38]. The sequence thus appears ideally suited to transmitting mechanical signals to the binding interface at low force.

### A 20–66 Cross Link within the DNase Domain of E9 Recovers Co-operativity

The close equivalence of k^0F^_off_ and k^i^_off_ of E9:Im9 upon cross-linking the N-terminal region of E9 to the remainder of this globular protein suggests that unbinding under ambient conditions and that induced by force occur by the same pathway. In this case, co-operativity between binding hot-spots on the immunity protein helices II and III is restored and mutation of residues in either helix should yield changes in the rate-limiting outer barrier that correlate with the change in affinity for that complex. Analysis of 3_20–66_:81 E9:immunity protein complexes that vary in their binding affinity from the tightly bound Im9 (K_d_ = 1.6×10^−14^ M), through Im2(D33A) (K_d_ = 1.0×10^−9^ M), to the weakly bound Im2 (K_d_ = 1.5×10^−7^ M) each yield k^0F^_off_ values close to the previously measured ensemble k^i^_off_ values (Figures 7 and S10). Together, these data provide further evidence that cross-linking switches the force-induced unbinding pathway (which involves remodelling of the E9 subunit within the complex) to a cooperative event that closely matches the thermally induced unbinding mechanism.

Only a single monotonic force regime is observed in the dynamic force spectrum of all complexes that contain a disulfide bridge, irrespective of their mechanical phenotype or K_d_ value that varies over seven orders of magnitude. This finding may reflect the absence of the second inner barrier (due to the co-operativity between each binding hotspot), or changes in the relative height of each barrier that results in an altered route of force propagation that moves the inner barrier to a loading rate beyond the dynamic range available to AFM. Irrespective of these changes to the energy landscape, the observation of k^0F^_off_ values that concur with previously determined k^i^_off_ values reveals that the rate-limiting step for the forced and thermally activated pathways is similar when the structural pliability of E9 is minimised by bolstering the E9 structure with disulfide cross-links. This observation has important implications for interpreting dynamic force experiments on proteins with mechanically labile structures and helps to explain the differences in off-rates frequently observed obtained by ensemble and DFS methods [39],[40]. The million-fold increase in k_off_ measured for E9:Im9 represents a striking example of this phenomenon.

## Discussion

### Dissociating Colicin:Immunity Proteins; A Novel Force-Induced Unbinding Event

The evolution of protein sequences has generated a rich repertoire of finely tuned protein-protein interactions whose binding affinities span ≈13 orders of magnitude [41]. Some complexes (barnase-barstar, for example) have evolved to bind tightly and to have a long lifetime (≈1.5 d at pH 8.0) [42]. Other, equally avid, complexes (for example SNARE complexes) need to dissociate more frequently for biological function [43]. Whilst altering protein sequence can modulate the binding affinity and the on- or off-rates of protein complexes, in some cases by many orders of magnitude [8],[44],[45], force-induced substrate remodelling offers further opportunities to tune the energy landscape of complex formation and dissociation. For example, interactions can become stronger (catch bonds) [46], or weaker (slip bonds) [27],[28] upon the application of force, and hidden epitopes required for binding can be exposed by forced unfolding (cryptic motifs) [31]. In the case of cell-cell adhesion mediated by protein-protein interactions, combinations of these have also been identified [47].

Here we have shown a striking example of how force-induced substrate remodelling can modulate complex stability (Figure 10B and 10C). At low loading rates and forces (<20 pN [48],[49]), highly avid cognate E9:immunity protein complexes dissociate in tens of milliseconds. Such lifetimes are ≈10^6^-times shorter than the thermally induced off-rate for the same complex (4.1 d) revealing a remarkable sensitivity of lifetime to force. These force-induced lifetimes are commensurate with the timescale for colicin intoxication of bacteria.

By altering the points of immobilisation and introducing disulfide cross-links at different locations, we identify the N-terminal region of E9 as a force transducer and suggest residues 21–30 as the location of the allosteric effector of force-triggered dissociation. The N-terminal region of E9 is highly conserved with a high content of Ala and Gly residues that render this region of the protein conformationally pliable. Such a sequence provides the ideal circuitry to relay a conformational trigger to the allosteric switch that lies close to the protein complex interface. This conformational rearrangement results in contour lengths at dissociation of all the complexes that are greater than expected (Figure S11; Text S1). This could reflect a degree of local unfolding in one or both proteins involved in the complex, or could result from deformation/elongation of the intact complex under force application prior to its dissociation.

In this study we have used an AFM to apply a stimulus to trigger remodelling of the E9:Im9 interface. In vivo, this triggering force may be driven by conformational re-arrangements caused by changes in the environment or by changes in other domains of the colicin that are transmitted to the DNase domain. Indeed, introduction of a disulfide cross-link across the N- and C-terminal regions of the R-domain of colicin, which is N-terminal to the nuclease domain (Figure 1B) prevents immunity protein release upon translocon formation [15]. In addition to local conformational changes upon formation of the translocon complex, induced conformational changes may drive immunity protein dissociation by (i) differential rates or extent of diffusion of the inner and outer membranes (or protein domains within these) [20] that are linked by the docked colicin:BtuB:OmpF:TolB translocon, or (ii) an energised motor-like function of TolQRA domains on the inner membrane. However, other stimuli may also result in the remodelling of the allosteric trigger. For example, facile dissociation of a colicin:immunity protein complex has also been reported for E3:Im3. In this case, binding of the complex to a strong anion exchange resin was suggested to induce conformational changes in the immunity protein that resulted in colicin release [50]. The responsiveness of E9 to its environment has been further demonstrated by the observation that insertion into a negatively charged membrane (required for colicin intoxication) is prevented by introduction of one the disulfide linkages (20–66) that we show here to prevent E9 remodelling.

### Protein Remodelling as a Universal Modulator of Protein Affinity

In addition to its biological implications for colicin intoxication, our study provides direct experimental evidence that force can induce changes in the energy landscape measured by dynamic force spectroscopy using the AFM and, for E9:Im9, provides a mechanism by which this occurs. The data show that, in addition to tilting of a “zero force” landscape as predicted and quantified by Bell, force can re-sculpt the underlying energy landscape. For E9:Im9 dissociation, we show that these changes allow facile dissociation of an avid complex at low forces. For example at 25 pN the dissociation rates of wild-type and cross-linked E9:Im9 complexes are 163 s^−1^ and 1.8×10^−4^ s^−1^. The ability to re-sculpt the energy landscape by force provides biology the opportunity to break apart highly avid complexes in the absence of a direct source of energy. In support of this, discrepancies between k^i^_off_ and k^0F^_off_ are observed for many complexes [39],[40] but, in contrast to the 10^6^-fold difference in off-rates observed for E9:Im9, these differences are typically relatively small (10^2^ at most). This suggests that colicin sequence and structure have evolved to enable triggered unbinding that is required for their biological function. In rare cases, such as the dissociation of an antigen from a kinetically and mechanically stable single-chain antibody (an immunoglobulin-like domain) excellent agreement between off rates is observed between ensemble and dynamic force spectroscopy methods [28]. This study, together with the identity of k^0F^_off_ and k^i^_off_ for the dissociation of Im9 from disulfide bridged E9 variants (residues 13 and 117 or 20 and 66) adds further support that conformational remodelling can drive dissociation in vivo. The remodelling force could be generated in many ways, such as by energy-dependent remodelling enzymes (AAA+ proteins, for example), by the binding of new ligands leading to changes in the dynamics or conformation of the complex, or by changing the chemical environment.

The force-induced switching between populations of protein complexes with distinct mechanical properties has been observed previously for the nuclear transport complex Ran:importin β [51] and subunits of von Willebrand factor involved in blood clotting [52]. While the mechanism underlying the force switch varies in these two cases, the application of force results in a switch to a more force resistant slip “bond” or, for von Willebrand factor, to a flex “bond.” (Note: these are not single bonds but a series of non-covalent interactions). For E9:Im9 the situation is reversed in that force induces a transition from a high resistance scenario (low k_off_) to a low force resistance slip bond (high k_off_). This is akin to a trip wire (a “trip bond”), whereby small forces trigger the remodelling of an interface that is very stable in the absence of force. The identification of a trip bond thus adds to the repertoire of behaviour of biomolecules under force that has emerged over the last decade [30],[46],[47],[52],[53] and provides a mechanism to explain the discrepancy in off-rates often observed between ensemble and DFS measurements. For colicin function, the force response of a trip bond meets the seemingly mutually exclusive requirements to provide long term protection to the host, yet permit the facile dissociation of immunity protein that is required for cell invasion of its competitors.

## Materials and Methods

### Protein Construction and Purification

Triple cysteine variants of E9 were designed using “Disulfide by design” software [54]. All proteins were created and purified as described previously [14].

### Surface Functionalisation

Silicon substrates were first cleaned by sonication in chloroform for 30 min and silicon nitride AFM cantilevers were cleaned by rinsing with chloroform for a minimum of 5 min. The substrates and AFM probes were then exposed to UV radiation (254 nm) for 30 min. Following this, surfaces to be functionalized were held under vacuum in the presence of 80 µl (3-aminopropyl)triethoxysilane (APTES) and 20 µl of N,N-diisopropylethylamine (DIPEA) for a period of 2 h. After this time the APTES and DIPEA were removed and the treated surfaces were left to cure under a nitrogen atmosphere for 24 h. These aminosilinated surfaces were then reacted with a heterofunctional PEG linker (NHS-(PEG)_n_-maleimide [*n* = 4 or 12, Thermo Scientific]) by adding 15 µl of 250 mM PEG linker in DMSO to 1 ml of chloroform in which the surfaces were incubated for 1 h. After functionalization with the PEG linker, the surfaces were washed using chloroform, dried under nitrogen and held under PBS until required. To avoid hydrolysis of the maleimide groups, functionalized surfaces were used within 1 h of their preparation. When required, functionalised surfaces and AFM probes were incubated with protein (at a concentration of 1 mgml^−1^ in PBS which, is in excess with respect to maleimide groups on the surfaces) for 30 min and then washed with PBS.

### Force Spectroscopy

All AFM measurements were conducted on an Asylum MFP-3D microscope using Si_3_N_4_ cantilevers with nominal spring constants of either 30 or 100 pNnm^−1^ (Bruker MLCT). For each cantilever used, the spring constant was determined using the thermal method [55],[56] via inbuilt fitting software. Retraction velocities of 200–8,000 nms^−1^ were employed for dynamic force spectroscopy analysis. For velocities between 200 and 5,000 nms^−1^ a PEG linker composed of 12 monomers was used, and for retraction velocities of 8,000 nms^−1^ a shorter PEG linker (four monomers) was used in order to increase the loading rate that could be applied. All experiments were conducted under PBS at 25°C. For retraction velocities less than 5,000 nms^−1^, 30 pNnm^−1^ nominal spring constant cantilevers were employed. At retraction velocities greater than 5,000 nms^−1^, 100 pNnm^−1^ nominal spring constant cantilevers (which have a smaller cross section) were used in order to reduce the hydrodynamic drag experienced by the cantilever, which becomes significant for the 30 pNnm^−1^ cantilevers at retraction velocities above 5,000 nms^−1^. A minimum of three separate functionalized AFM tips and surfaces were used for the collection of each dynamic force spectrum measured.

### Measurement of Ensemble Dissociation Rate Constants (k^i^_off_)

Size exclusion chromatography (SEC) was used to quantify the release of E9 DNase into solution over time from an E9 DNase:Im9 complex incubated in the presence of excess full length colicin E9. This procedure was used to measure k^i^_off_ for both E9 (S3C):Im9 (S81C) derivatised with a PEG linker and E9_20–66_ domains (see text) in complex with Im9, under conditions identical to those employed for the DFS experiments. E9 and Im9 were derivatised with methyl-(PEG)_12_-maleimide ((MM(PEG)_12_), Thermo Scientific) by incubation of the protein with a 20-fold molar excess of MM(PEG)_12_ overnight at room temperature in 25 mM Tris.HCl buffer, 1 mM MgCl_2_ (pH 7.5). Following this, derivatised protein was separated from un-labelled protein and excess MM(PEG)_12_ by size exclusion chromatography.

E9 DNase domains were first incubated with Im9 at a molar ratio of 1∶2 in order to form the E9 DNase:Im9 complex. The E9 DNase:Im9 complex was then purified from the excess Im9. A 25-µM solution of the E9 DNase:Im9 complex and 125-µM of full length colicin E9 in PBS buffer (pH 7.3), 0.01% (w/v) azide and a protease inhibitor cocktail (set III, EDTA free, Calbiochem) was then incubated for different lengths of time. Samples were removed at various times between 0 and 144 h and analysed via SEC. The intensity of the elution peak that corresponded to the free E9 DNase domain (competed from the complex by the addition of excess of full length colicin E9) was quantified as a function of time to calculate an apparent dissociation rate constant (k^i^_off_). An example dataset is shown in Figure S8.

### Nuclease Activity Assay

E9 nuclease activity was assessed by monitoring the conversion of supercoiled DNA into other forms upon addition of E9 DNase domain (E9 (S108C) or E9 (S108C) derivatised with MM(PEG)_12_ as described above. DNA that was predominantly in the supercoiled conformation was isolated using a Hi-speed midi prep kit (Qiagen) at 4°C as described [57]. Nuclease activity was measured by addition of 30 nM E9 (final concentration) to purified DNA at a concentration of 50 µg/ml in 25 mM Tris.HCl buffer containing 1 mM MgCl_2_ (pH 7.5) in the presence or absence of 50 nM Im9. The reaction was arrested by adding 10 µl of this solution to 5 µl of solution containing 20 mM EDTA and agarose gel electrophoresis loading dye. Multiple time points were taken and the presence of supercoiled, linear, and open circular DNA was assessed by visualisation using agarose gel electrophoresis.

## Supporting Information

Figure S1 **Depiction of the covalent chemical attachment method for tethering proteins containing unique solvent exposed cysteine residues to silicon surfaces and AFM cantilevers.**
(TIF)

Figure S2 **Examples of force-distance profiles for E9:Im9 dissociation events.** (A) Detail for a single event. Dissociation force and linker stiffness at rupture are indicated. Loading rate at rupture is given by the product of the linker stiffness at rupture and the retraction velocity. WLC fit (red) to experimental data (blue), in this case obtained for E9:Im9 (3_20–66_:81) at a retraction velocity of 1,000 nms^−1^. (B) Example dissociation events are shown for (i) 108:81, (ii) 3:81, (iii) 3_20–66_:81, and (iv) 3_13–117_:81 E9:Im9 interactions at four different retraction velocities (200, 500, 1,000, and 3,000 nms^−1^). Red lines represent a WLC fit to the data. The fitted contour length for each fit is inlaid. Dashed vertical lines represent the average observed contour length for the whole dataset (see Figure S11).
(TIF)

Figure S3 **Schematic representation of considerations for determining the observed contour length at dissociation.** For single molecule unbinding experiments of protein-protein interactions, the observed contour length is dependent upon the attachment location on both the tip and the surface. The maximum observable contour length (red line) corresponds to the interaction of Im9 immobilised onto the tip apex by a linker and an E9 molecule attached to the substrate (via a linker) directly beneath the tip (situation 2). Attachment of Im9 (via a linker) distal to the tip apex results in shorter observed contour lengths than expected (blue line, situation 1).
(TIF)

Figure S4 **Comparison of simulated contour length distributions to real data.** (A) Simulated contour length distribution for the 3:81 pulling geometry where L_c_^max^ = 17.96 nm (green dashed line). (B) Real data for comparison to simulated data for the 3:81 pulling geometry both with and without the disulfide cross links used in this study.
(TIF)

Figure S5 **Optimisation of parameters used in data filtering software.** Force frequency histograms are shown for three retraction velocities (columns) extracted using different threshold values input into the analysis software (rows). Top row: forces due to thermal noise. Middle row: the highest force events due to thermal noise used to determine the force sensitivity of the experiment. Bottom row: dataset analysed with threshold values used throughout data analysis where only events above the previously determined thermal noise limit of the instrument are detected.
(TIF)

Figure S6 **Comparison of single and multiple Gaussian fits to unbinding force frequency distributions.** For each E9:immunity protein pair shown (red, E9:Im9 (3:81); dark yellow, E9:Im2(D33A) 3:81; and blue, E9_20–66_:Im9 3:81) force-frequency distributions were fitted to either a single (top row) or multiple Gaussian distribution(s) (bottom row, sum of the distributions are shown as a dashed green line) for three different pulling velocities (200, 1,000, and 5,000 nms^−1^). Inlaid forces are the modes of the fit. For multiple Gaussian fits, the mode value of the first, highest amplitude Gaussian is almost identical to the mode value for a single Gaussian fit.
(TIF)

Figure S7 **Fourier transform ion cyclotron mass spectrum of the triple cysteine mutant E9 3_20–66_.** Magnified mass spectrum for the 9+ charge state of E9 3_20–66_. The full mass spectrum is also shown (inset). The data demonstrate the absence of any E9 3_20–66_ in its reduced form. Note: the second low intensity distribution reveals the presence of low levels of dimers. These cannot be immobilised onto the AFM substrate. The theoretical mono-isotopic masses for E9 3_13–117_, E9 3_20–66_, and E9 3_31–122_ containing a single disulfide bridge are 15,127.69, 15,055.67, and 15,141.67 Da, respectively. The masses observed in this experiment are 15,127.7, 15,055.7, and 15,141.7, respectively.
(TIF)

Figure S8 **Determination of the solution dissociation rate for the E9_20–66_:Im9 complex.** Size exclusion chromatogram (left) showing the increase in free E9 (in this case E9_20–66_) over time as it is chased off Im9 by an excess of full length ColE9. In these experiments, k^i^_off_ was determined (right) by measuring the amplitude of the peak in the chromatogram that corresponds to free E9. The data were then fitted to a single exponential (dotted line) in order to determine k^i^_off_ for the E9:Im9 complex (red points, E9_20–66_:Im9; blue points E9(S3C):Im9(S81C) derivatised with methyl-(PEG)_12_-maleimide.
(TIF)

Figure S9 **The redox status of E9 determines the mechanical phenotype of E9:Im9 (3_20–66_:81).** Upon addition of 4 mM DTT reduction of the disulfide bond switches the mechanical phenotype of E9:Im9 (3_20–66_:81) from strong, with a single force regime observable in the dynamic force spectrum (oxidised, open circles) to weak with a two force-regime dynamic force spectrum identical to the wild-type complex (reduced, closed circles). For reference, best-line fits of the dynamic force spectrum data across the dynamic range of the instrument are shown for E9:Im9 (3:81) and E9:Im9 (3_20–66_:81) obtained under standard conditions (PBS, 25°C). Arrows indicate the change in the unbinding force of E9:Im9 (3_20–66_:81) that takes place upon DTT addition at retraction velocities of 500 and 3,000 nms^−1^.
(TIF)

Figure S10 **Comparison of the dynamic force spectrum for cognate and non-cognate immunity proteins bound to E9 (3_20–66_).** In the presence of a disulfide bridge between residues 20 and 66, k^0F^_off_ values extracted using the Bell-Evans model (listed above the data) accord (within a factor of 10) with ensemble off-rates (k^i^_off_ = 1.83 [±0.09]×10^−6^ s^−1^, 0.054 [±0.003] s^−1^, and 0.88 [±0.04] s^−1^ for E9:Im9, E9:Im2[D33A], and E9:Im2, respectively) [7],[8].
(TIF)

Figure S11 **Remodelling of E9 does not significantly increase the end-to-end length of the complex before dissociation.** Coloured bars show the observed contour lengths (modal averages for all data obtained for each pulling geometry and disulfide cross-link combination). The theoretical L_c_^max^ values and the most commonly observed simulated contour length using the same L_c_^max^ values are shown as dashed and dotted lines, respectively. See Text S1 for a description of the model. L_c_^max^ was calculated as the sum of linker lengths and the through-space distance between pulling residues from the crystal structure (PDB file:1EMV [5]). All averages for observed contour lengths are slightly greater than the most commonly observed contour length found using the model suggesting that the protein complex is deformed/elongated under force application before unbinding occurs.
(TIF)

Table S1 **Summary of DFS data.** Values of k^0F^_off_ and x_u_ measured by DFS in this study. For datasets where two free energy barriers are detected over the loading rate range investigated in this work, k^0F^_off_ and x_u_ are stated for both the rate-limiting (outer) and the intermediate (inner) barrier. Errors on the event probability are based on the standard deviation of measured values. Errors for k^0F^_off_ and x_u_ were calculated using a Jackknife method.
(DOC)

Text S1 **Modelling contour length distributions.**
(DOCX)

Text S2 **Force curve analysis.**
(DOCX)

Text S3 **Determination of force sensitivity.**
(DOCX)

Text S4 **Measuring the most probable force at rupture.**
(DOCX)

Text S5 **Measurement of dynamic force spectra and the Bell parameters k^0F^_off_ and x_u_.**
(DOCX)

## Abbreviations

AFM: atomic force microscope
PBS: phosphate buffered saline
PEG: polyethylene glycol
PMF: proton motive force
