# Supplementary figures and images for: A Force-Activated Trip Switch Triggers Rapid Dissociation of a Colicin from Its Immunity Protein
Source: PLoS Biol. 2013 Feb 19;11(2):e1001489. doi: 10.1371/journal.pbio.1001489 (PMC3576412; doi:10.1371/journal.pbio.1001489)

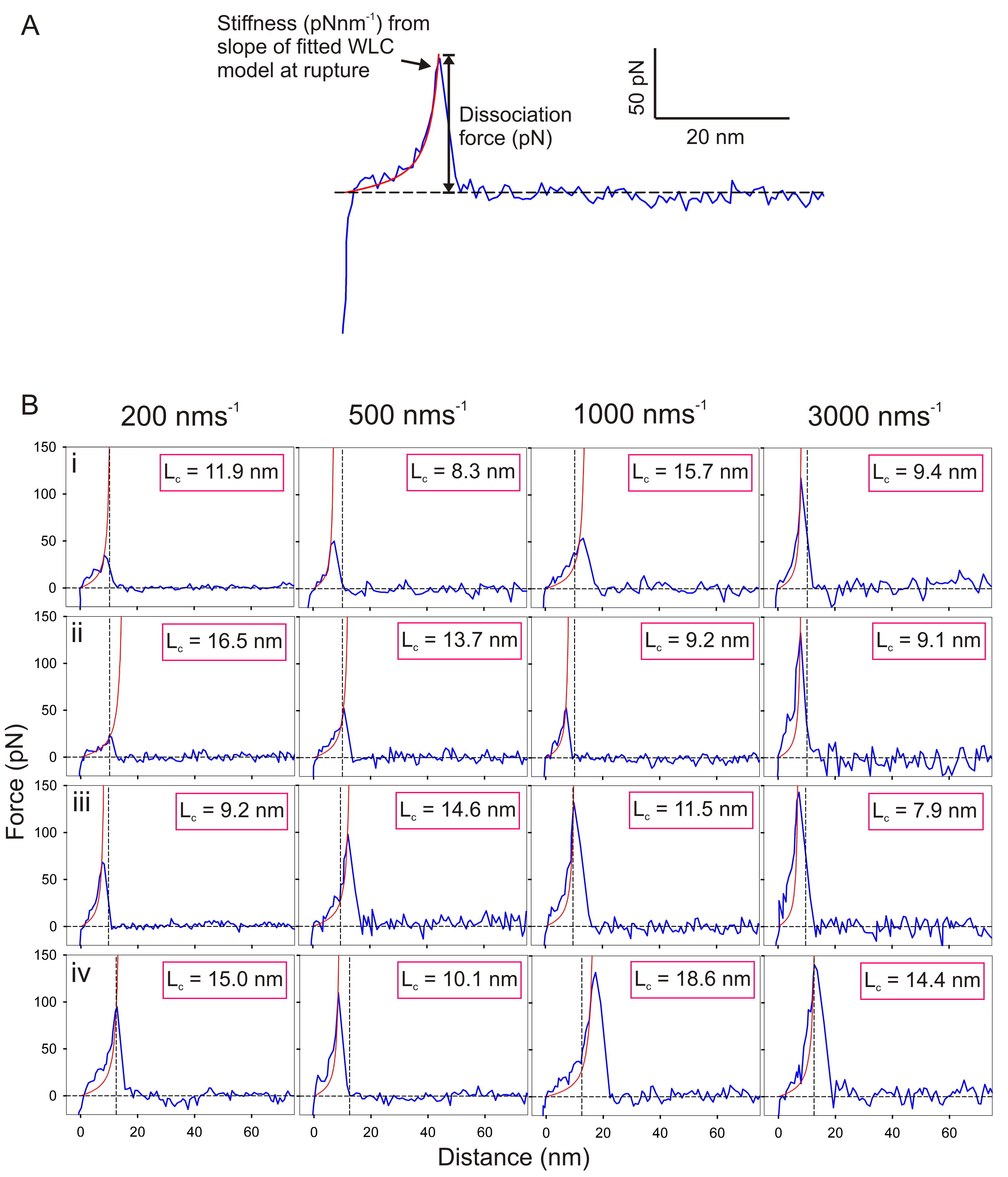

Supplement: Figure S2 — Examples of force-distance profiles for E9:Im9 dissociation events. (A) Detail for a single event. Dissociation force and linker stiffness at rupture are indicated. Loading rate at rupture is given by the product of the linker stiffness at rupture and the retraction velocity. WLC fit (red) to experimental data (blue), in this case obtained for E9:Im9 (320–66:81) at a retraction velocity of 1,000 nms−1. (B) Example dissociation events are shown for (i) 108:81, (ii) 3:81, (iii) 320–66:81, and (iv) 313–117:81 E9:Im9 interactions at four different retraction velocities (200, 500, 1,000, and 3,000 nms−1). Red lines represent a WLC fit to the data. The fitted contour length for each fit is inlaid. Dashed vertical lines represent the average observed contour length for the whole dataset (see Figure S11). (TIF) [file pbio.1001489.s002.tif]
