# Supplementary material for: A Force-Activated Trip Switch Triggers Rapid Dissociation of a Colicin from Its Immunity Protein
Source: PLoS Biol. 2013 Feb 19;11(2):e1001489. doi: 10.1371/journal.pbio.1001489 (PMC3576412; doi:10.1371/journal.pbio.1001489)
Supplement: Table S1 — Summary of DFS data. Values of k0F off and xu measured by DFS in this study. For datasets where two free energy barriers are detected over the loading rate range investigated in this work, k0F off and xu are stated for both the rate-limiting (outer) and the intermediate (inner) barrier. Errors on the event probability are based on the standard deviation of measured values. Errors for k0F off and xu were calculated using a Jackknife method. (DOC) [file pbio.1001489.s012.doc]

| Interaction  tested | Mean number of unbinding events observed per retraction velocity | Event probability (%) | Outer barrier | | Inner barrier | |
| --- | --- | --- | --- | --- | --- | --- |
|  |  |  | k^0F^_off_ (s^-1^) | x_uo_ (Å) | k^0F^_off_ (s^-1^) | x_ui_ (Å) |
| E9:Im9 (3:38) | 917 | 11.7 ± 3.3 | 6.7 ± 1.1 | 5.0 ± 0.2 | 60 ± 34 | 1.1 ± 0.3 |
| E9:Im9 (3:81) | 632 | 11.5 ± 3.1 | 4.9 ± 1.3 | 5.8 ± 0.4 | 50 ± 17 | 0.86 ± 0.16 |
| E9:Im9(V34A) (3:81) | 536 | 5.2 ± 1.0 | 2.2 ± 1.2 | 6.6 ± 1.0 | 97 ± 19 | 1.1 ± 0.1 |
| E9:Im9 (30:81) | 757 | 7.1 ± 1.9 | 0.42 ± 0.33 | 4.5 ± 0.4 | 112 ± 48 | 1.3 ± 0.2 |
| E9:Im9 (108:38) | 731 | 12.9 ± 2.0 | 0.51 ± 0.35 | 8.1 ± 0.9 | 136 ± 93 | 0.47 ± 0.02 |
| E9:Im9 (108:81) | 896 | 10.3 ± 3.2 | 1.5 ± 1.2 | 5.9 ± 0.8 | 45 ± 3 | 0.72 ± 0.02 |
| E9:Im2(D33A) (108:81) | 729 | 9.9 ± 2.9 | 8.3 ± 0.9 | 3.5 ± 0.07 | - | - |
| E9:Im9 (3_20-66_:38) | 650 | 8.3 ± 1.2 | (4.0 ± 3.2) × 10^-5^ | 6.8 ± 0.3 | - | - |
| E9:Im9 (3_20-66_:81) | 614 | 7.7 ± 2.5 | (1.4 ± 1.2) × 10^-6^ | 8.1 ± 0.4 | - | - |
| E9:Im2(D33A) (3_20-66_:81) | 615 | 9.2 ± 2.5 | 0.52 ± 0.36 | 4.3 ± 0.4 | - | - |
| E9:Im9 (3_13-117_:81) | 415 | 3.9 ± 0.6 | (4.6 ± 4.2) × 10^-6^ | 8.0 ± 0.2 | - | - |
| E9:Im9 (3_31-122_:81) | 594 | 5.7 ± 1.0 | 5.7 ± 2.0 | 5.0 ± 0.5 | - | - |
| E9:Im9 (108_20-66_:81) | 650 | 6.3 ± 1.0 | (5.3 ± 3.8) × 10^-4^ | 6.4 ± 0.3 | - | - |
| E9:Im2(D33A)  (3:81) | 482 | 4.2 ± 1.0 | 7.6 ±1.8 | 4.3 ± 0.3 | 142 ± 10 | 1.1 ± 0.03 |
| E9:Im2 (3_20-66_:81) | 769 | 7.2 ± 0.9 | 7.9 ± 1.0 | 3.8 ± 0.1 | - | - |

**Table S1: summary of DFS data.** Values of k^0F^_off_ and x_u_ measured by DFS in this study. For datasets where two free energy barriers are detected over the loading rate range investigated in this work, k^0F^_off_ and x_u_ are stated for both the rate-limiting (outer) and the intermediate (inner) barrier. Errors on the event probability are based on the standard deviation of measured values. Errors for k^0F^_off_ and x_u_ were calculated using a Jackknife method.
