# Supplementary material for: A Force-Activated Trip Switch Triggers Rapid Dissociation of a Colicin from Its Immunity Protein
Source: PLoS Biol. 2013 Feb 19;11(2):e1001489. doi: 10.1371/journal.pbio.1001489 (PMC3576412; doi:10.1371/journal.pbio.1001489)
Supplement: Text S1 — Modelling contour length distributions. (DOCX) [file pbio.1001489.s013.docx]

**Text S1: Modelling contour length distributions.**

In experiments where a homopolymeric protein construct is mechanically unfolded, a reproducible interpeak distance between each unfolding event is observed that is related to the number of residues within the globular protein that have been unfolded [1]. However, in the case of observing single rupture events between interacting proteins (as in this work) only a single event is present and the tip-substrate separation distance at rupture is variable. Furthermore the observed contour length for single rupture events is generally below that which may be expected (L_c_^max^, Figure S3) based on the combined linker and protein domain lengths. If we limit ourselves to only analysing events that correspond to L_c_^max^ then a large proportion of otherwise useable force curves are discarded as events that occur at L_c_^max^ correspond to the highly unlikely situation of one linker attachment position at the apex of the AFM tip and another directly beneath it. For these reasons a Monte Carlo algorithm was developed to generate simulated contour length distributions that would be expected for particular linker and protein dimensions which were then compared to experimental data.

If the binding partner that is immobilised on the AFM cantilever (Im9 in this case) is attached at some location other than the tip apex then contour lengths at rupture shorter than L_c_^max^ will be observed (Figure S3). Additionally, the surface area available for derivatisation with linkers increases with increasing distance from the tip apex. The probability of observing contour lengths shorter than L_c_^max^ should thus be higher than events at exactly L_c_^max^. In addition to the effect of protein attachment location, the length of the linkers used will influence the observed distribution of contour lengths. This is because the probability density function for the end-to-end distance within the conformational ensemble of the linkers (which is dependent on their length) will determine the volume of space explored by the protein around its linker attachment position. For this study, the linkers were assumed to be under good solvent conditions and have a distribution of chain end-to-end distances corresponding to a self-avoiding random walk.

For the E9:Im9 system under investigation in this work, force application leads to some degree of deformation of the complex before rupture. In this case some extra length in addition to the sum of the linkers and the through space distance between pulling residues (from crystal structure PDB file:1EMV) will need to be considered in order to match well with experimental results. For single molecule forced unbinding experiments on the E9:Im9 system where each protein is attached by linkers of length 6.62 nm (as in the large majority of this work) and where each protein behaves as a “rigid body” then the L_c_^max^ value should be largest for the 108:81 pulling geometry (18.23 nm) and lowest for the 3:38 pulling geometry (15.97 nm).

Data was simulated using different values of L_c_^max^. One example of a simulated contour length distribution is shown below (Figure S4A). This shows the expected distribution when a value for L_c_^max^ of 17.96 nm (corresponding to the 3:81 pulling geometry) is used. Each simulated distribution shows a peak at contour lengths below L_c_^max^ with a sharp cut-off at lower contour lengths. Comparison of real data (Figure S4B) to simulated data for the same pulling geometry (3:81, Figure S4A) reveals reasonably good qualitative agreement with the shape of the distributions and a small but consistent underestimate of the most probable contour length (peak in distribution) relative to the expected data. See Figure S11 for quantification of simulated and experimentally observed contour lengths.

**References**

1. Carrion-Vazquez M, Oberhauser AF, Fowler SB, Marszalek PE, Broedel SE, et al. (1999) Mechanical and chemical unfolding of a single protein: A comparison. Proc Natl Acad Sci U S A 96: 3694-3699.
