# Supplementary material for: A Force-Activated Trip Switch Triggers Rapid Dissociation of a Colicin from Its Immunity Protein
Source: PLoS Biol. 2013 Feb 19;11(2):e1001489. doi: 10.1371/journal.pbio.1001489 (PMC3576412; doi:10.1371/journal.pbio.1001489)
Supplement: Text S2 — Force curve analysis. (DOCX) [file pbio.1001489.s014.docx]

**Text S2: Force curve analysis.**

Approximately 1 in 10 approach-retract cycles resulted in a true unbinding event. Consequently, an automated analysis script [1,2] (run in Igor Pro) was employed for high throughput filtering and analysis. After identifying the hard contact and the baseline (zero force regime) from which forces and distances are measured, the automated analysis process calculates the rate of change of the force response signal from the force-distance curve. When an unbinding event is encountered, there is a large change in the force response and hence a peak in the observed rate of change of the force response. If this rate of signal change goes above, then below certain pairs of threshold values set by the user (see below) then the event is detected and analysed using the automated process.

All detected events are then automatically fit to the worm-like chain (WLC) model [3] where the persistence length is fixed at a value preset by the user (4 Å). First, detected events were inspected in order to determine any filtering parameters to be used so as to ensure only specific single molecule dissociation events are analysed. The tip-sample separation distance for each detected event was found to be less than or equal to the length of the sum of the linkers and protein domain lengths in >80 % of cases and less than or equal to this length plus 5 nm in >95% of cases (note that this percentage is the fraction of force curves displaying any event above the noise of the experiment and not all collected force curves). Furthermore fewer than 0.5% of force curves showed more than a single detected event. This indicates that there are no detectable unfolding events for either protein domain prior to dissociation of the bound complex. If this were the case then force curves containing multiple events would frequently be observed where the first event would correspond to unfolding and the final event to complex dissociation. Therefore we observe no direct evidence of unfolding occurring prior to dissociation. The proportion of force curves displaying multiple events (0.5%) is consistent with the probability of multiple binding events occurring where the probability of single binding events is 7.1%. Detected events for tip-sample separation values of less than 5 nm were in general found to be consistent with non-specific tip-sample interactions. Namely they displayed a linear force-distance profile (single molecule events should display WLC like behaviour where the force-distance profile is not linear). These observations allowed optimisation of filtering parameters so that only true single molecule events are analysed. Force-extension profiles to be binned for further analysis satisfied the following criteria: (i) had a rupture force larger than the thermal noise of the experiment (see Text S3); (ii) had a distance to the rupture event from the hard tip-surface contact that is within a certain range governed by the length of linkers used and the through-space distance between extension points of the interacting proteins (lower limit of 5 nm, upper limit 32.5 or 40 nm for PEG_4_ and PEG_12_ linkers respectively); (iii) displayed only a single unbinding event and (iv) had a force-extension profile with a good fit to the WLC model. For many of these criteria the use of PEG linkers facilitated the identification of true rupture events as their force-extension behaviour (which dominates the profiles) are well known.

Loading rates were calculated by fitting a WLC model, equation S1) to the rising edge of each unbinding profile when plotted as force versus tip-sample separation. The instantaneous gradient of this fit at rupture (df/dx) was calculated by inserting the derived contour length and extension at rupture into a differentiated form of the same equation (equation S2). The loading rate at rupture was then obtained by multiplying this value by the retraction velocity.

**Equation S1**

**Equation S2**

Where; f_wlc_ is the force acting on the WLC, b is the persistence length, l is the contour length, x is the extension, k_B_ is the Boltzmann constant and T is the temperature.

**References**

1. Fuhrmann A, Anselmetti D, Ros R, Getfert S, Reimann P (2008) Refined procedure of evaluating experimental single-molecule force spectroscopy data. Phys Rev E 77: 031912.
2. Fuhrmann A, Schoening JC, Anselmetti D, Staiger D, Ros R (2009) Quantitative analysis of single-molecule RNA-protein interaction. Biophys J 96: 5030-5039.
3. Marko JF, Siggia ED (1995) Stretching DNA. Macromolecules 28: 8759-8770.
