# Supplementary material for: A Force-Activated Trip Switch Triggers Rapid Dissociation of a Colicin from Its Immunity Protein
Source: PLoS Biol. 2013 Feb 19;11(2):e1001489. doi: 10.1371/journal.pbio.1001489 (PMC3576412; doi:10.1371/journal.pbio.1001489)
Supplement: Text S3 — Determination of force sensitivity. (DOCX) [file pbio.1001489.s015.docx]

**Text S3: Determination of force sensitivity.**

Data from an arbitrary set of E9:Im9 forced unbinding experiments at three different retraction velocities (200, 1000 and 5000 nms^-1^) were taken and analysed with the analysis software using different pairs of threshold values.

Data were first analysed using threshold values that were very low and close to each other permitting the detection of small forces by the software. Any event or deviation away from the zero force baseline is detected and analysed (Figure S5, top row) allowing detection AFM cantilever fluctuations due to thermal motions in the surrounding buffer. In this case only events ≥ 40 nm from the surface were investigated to avoid the detection of real E9:Im9 unbinding events. The analysis software only detects the final event that satisfies the criteria governed by the threshold values and so the highest forces detected with these lowest threshold values (Figure S5, top row) do not necessarily represent the largest forces experienced by the AFM cantilever due to thermal fluctuations. To find the force sensitivity trial and error with different threshold values was employed in the analysis (again only for events ≥ 40 nm of tip sample separation). At threshold values of 72 and 54 only the highest force thermal events were detected (Figure S5, middle row). These events represent the force sensitivity of the experiment (18 pN) as there were zero events at forces higher than this.

Optimisation of threshold parameters

To determine optimum threshold values for data filtering and analysis of E9:immunity protein unbinding events, force-extension profiles that contained unbinding events at tip-sample separation distances (measured by WLC fits) in the range expected for the linkers and proteins used were analysed. Threshold values were iteratively increased from the values used to determine the thermal noise (72 and 54) until no events were observed below the previously determined force sensitivity of the experiment (18 pN). These threshold values (80 and 56, Figure S3, bottom row) were used throughout subsequent data analysis.
