# Supplementary material for: A Force-Activated Trip Switch Triggers Rapid Dissociation of a Colicin from Its Immunity Protein
Source: PLoS Biol. 2013 Feb 19;11(2):e1001489. doi: 10.1371/journal.pbio.1001489 (PMC3576412; doi:10.1371/journal.pbio.1001489)
Supplement: Text S4 — Measuring the most probable force at rupture. (DOCX) [file pbio.1001489.s016.docx]

**Text S4: Measuring the most probable force at rupture.**

Single Gaussian distributions were fit to unbinding force histograms in order to determine the most probable force at rupture for each retraction velocity investigated. Some distributions were found to be asymmetric with multiple high force events at approximately double or triple the most probable rupture force. These are thought to be due to the formation of multiple colicin:immunity protein complexes during a single approach-retract cycle [1-3].

To quantify the effect of fitting a single Gaussian to these data, the modes from these fits were compared to those obtained from the same data fitted to multiple Gaussian distributions. In these cases the mode of the second (and subsequent) Gaussians were set to be at least 150% of the first (or preceding) Gaussians. In all cases where such analysis was performed, the positions of the peak of the first Gaussian was found to vary by less than a bin width of the histogram when fitted to single or multiple Gaussians (Figure S6). Determination of most probable rupture force via this method has previously been shown to yield accurate estimates for k^0F^_off_ [4].

**References**

1. Kudera M, Eschbaumer C, Gaub HE, Schubert US (2003) Analysis of metallo-supramolecular systems using single-molecule force spectroscopy. Adv Funct Mater 13: 615-620.
2. Maki T, Kidoaki S, Usui K, Suzuki H, Ito M, et al. (2007) Dynamic force spectroscopy of the specific interaction between the PDZ domain and its recognition peptides. Langmuir 23: 2668-2673.
3. Neundlinger I, Poturnayova A, Karpisova I, Rankl C, Hinterdorfer P, et al. (2011) Characterization of enhanced monovalent and bivalent thrombin DNA aptamer binding using single molecule force spectroscopy. Biophys J 101: 1781-1787.
4. Getfert S, Reimann P (2012) Hidden multiple bond effects in dynamic force spectroscopy. Biophys J 102: 1184-1193.
