# Supplementary material for: A Force-Activated Trip Switch Triggers Rapid Dissociation of a Colicin from Its Immunity Protein
Source: PLoS Biol. 2013 Feb 19;11(2):e1001489. doi: 10.1371/journal.pbio.1001489 (PMC3576412; doi:10.1371/journal.pbio.1001489)
Supplement: Text S5 — Measurement of dynamic force spectra and the Bell parameters k0Foff and xu. (DOCX) [file pbio.1001489.s017.docx]

**Text S5: Measurement of dynamic force spectra and the Bell parameters k^0F^_off_ and x_u_.**

A dynamic force spectrum reveals how the unbinding force varies as a function of natural log of the force loading rate at rupture (which is related to the retraction velocity). The DFS for each E9:immunity protein pair was thus measured by quantifying the most probable unbinding force and loading rate at rupture over the widest possible range of retraction velocities. To minimise error, datasets were obtained in triplicate for each retraction velocity and analysed separately.

For each dataset at each retraction velocity, the most probable unbinding force was found by measuring the mode of a single Gaussian fit (see Text S4) to an unbinding force-frequency histogram generated for each dataset. The most probable ln(loading rate at rupture) was obtained by measuring the mode of a single Gaussian fit to a ln(loading rate at rupture)-frequency histogram generated for each dataset. The mean values of unbinding force and ln(loading rate at rupture) from the triplicate datasets for all retraction velocities were then plotted against each other.

Values for k^0F^_off_ (the dissociation rate in the absence of force) and x_u_ (the distance along the free energy landscape from the ground state to transition state) were obtained by fitting the Bell-Evans [1] equation (equation 1, main text). Straight line fits to the DFS plotted as described above thus allow values of k^0F^_off_ and x_u_ to be extracted. Errors stated in Table S1 and throughout the text are calculated using a Jackknife method.

**References**

1. Evans E, Ritchie K (1997) Dynamic strength of molecular adhesion bonds. Biophys J 72: 1541-1555.
